# BCL-2 family deregulation in colorectal cancer: potential for BH3 mimetics in therapy

**DOI:** 10.1007/s10495-020-01601-9

**Published:** 2020-04-25

**Authors:** Prashanthi Ramesh, Jan Paul Medema

**Affiliations:** 1 Laboratory for Experimental Oncology and Radiobiology, Center for Experimental and Molecular Medicine, Cancer Center Amsterdam, Amsterdam UMC, University of Amsterdam, Meibergdreef 9, 1105 AZ Amsterdam, The Netherlands; 2 Oncode Institute, Meibergdreef 9, 1105 AZ Amsterdam, The Netherlands

**Keywords:** Apoptosis, BCL-2 family, BH3 mimetics, Colorectal cancer

## Abstract

Apoptosis is a form of programmed cell death that is essential for tissue homeostasis. De-regulation of the balance between proliferation and apoptosis contributes to tumor initiation. Particularly in the colon where apoptosis is a crucial process in intestinal turnover, inhibition of apoptosis facilitates transformation and tumor progression. The BCL-2 family of proteins are key regulators of apoptosis and have been implicated in colorectal cancer (CRC) initiation, progression and resistance to therapy. In this review we outline the current knowledge on the BCL-2 family-regulated intrinsic apoptosis pathway and mechanisms by which it is de-regulated in CRC. We further review BH3 mimetics as a therapeutic opportunity to target this pathway and evaluate their potential for CRC treatment.

## Introduction

Programmed cell death is an essential process regulating tissue homeostasis and stress response in many organisms. One of the most widely studied and well-characterized forms of programmed cell death is apoptosis, first described in the landmark study of Kerr et al. [1]. In their study they describe a unique morphology of dying cells which form membrane bound fragments that get phagocytosed by nearby cells. Further understanding of the pathway came from developmental studies conducted in the nematode *Caenorhabditis elegans* [2]. It is now well accepted that this process is tightly regulated by a set of proteins with specific interaction domains, most of which are conserved in mammals [2, 3]. In response to various signals, these proteins orchestrate a cascade of reactions that ultimately dictate the choice between life and death.

While apoptosis occurs in normal tissue development and regeneration, it can also be activated in response to stress signals such as nutrient deprivation, reactive oxygen species and excessive mitogenic signaling usually associated with cancer initiation [4]. Such signals lead to the activation of one of the two main apoptotic pathways—the extrinsic and intrinsic pathways. The extrinsic pathway is regulated by so-called death receptors such as TNFR, FAS, DR3/WSL. Upon ligand binding these receptors activate signaling cascades that result in caspase activation, which is instrumental in the execution of apoptotic cell death. In this review we focus on the role of the BCL-2 family of proteins in cancer and hence on the intrinsic or mitochondrial pathway of apoptosis, which is regulated by this family [5].

Apoptosis is a key cell death mechanism that can counteract tumor formation and growth and for this reason, is often de-regulated in various cancers [6]. Increased proliferation resulting from oncogenic mutations is facilitated by genetic and epigenetic alterations in apoptotic pathways that ultimately allow uncontrolled tumor growth. Homeostasis in the colon is tightly regulated by a balance between proliferation and apoptosis. Disruption of this balance is an integral step in CRC development and progression. In addition, an increased apoptotic threshold is often observed in CRC tumors which contributes to therapy resistance [7]. In this review we describe how the members of the BCL-2 family regulate apoptosis and how they often get de-regulated to enable CRC progression and chemo-resistance. We further assess the potential of BH3 mimetics—small molecule antagonists of anti-apoptotic BCL-2 family members—as a therapeutic strategy for targeting this pathway and inducing apoptosis in CRC tumors.

### The intrinsic apoptosis pathway

In the intrinsic apoptosis pathway, the BCL-2 family of proteins play a key role in determining the decision to undergo apoptosis. The first member of the BCL-2 family to be identified was the pro-survival B-cell lymphoma-2 (*Bcl-2*) gene, which was found to be frequently amplified in lymphomas by an oncogenic translocation [8, 9]. It was soon discovered that this protein was able to promote cancer cell survival by preventing apoptosis [10–12]. More than 15 members of this family have since been identified that can be segregated based on their apoptotic regulation as either pro- or anti-apoptotic [13–17]. Here we describe how these members interact to regulate mitochondrial permeabilization.

#### The pro- and anti- apoptotic BCL-2 proteins

All members of the BCL-2 family of proteins show homology in one or more of the four BCL-2 homology (BH) domains and are categorized based on the number of homology domains they possess [18]. The anti-apoptotic members all have four BH-domains and include BCL-2, BCL-XL, MCL-1, BCL-W and A1/BFL-1. Of these, the BH1, BH2 and BH3 domains contribute to the formation of a hydrophobic pocket in their tertiary protein structure. The pro-apoptotic proteins can be divided into two sub-groups: the BH3-only proteins and the effector proteins. The BH3-only proteins, thus named as they only show homology to the BH3 domain of BCL-2 include BIM, BAD, BID, PUMA, NOXA, BMF, HRK, BIK and others. The effector proteins such as BAX and BAK possess three to four BH domains and are able to form macropores in the mitochondrial outer membrane. BOK is another multi-BH3 domain effector protein that has pro-apoptotic functions, which some studies suggest to be independent of BAX and BAK [19–21]. The physical interaction between anti- and pro-apoptotic proteins is a result of the binding of the BH3 domain of the pro-apoptotic proteins to the hydrophobic clefts of the anti-apoptotic proteins [18, 22, 23].

#### Regulation of mitochondrial permeabilization

**Fig. 1:**
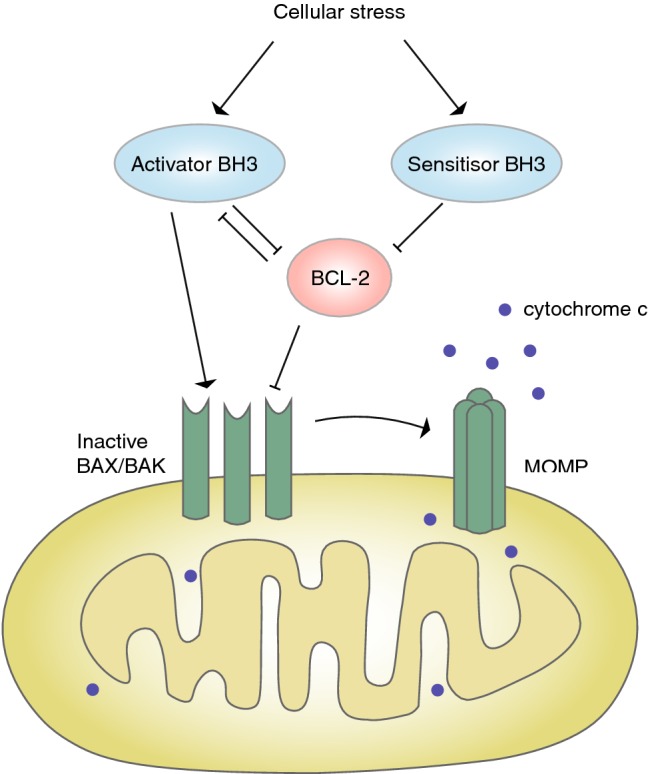
BCL-2 family protein interactions regulate mitochondrial outer membrane permeabilization (MOMP)

The vast majority of BAX and BAK molecules exist as inactive monomers (BAX) or homodimers (BAK) in an equilibrium between the cytosol and mitochondrial membrane [24–26]. BAX/BAK activation leads to the formation of pores that initiates the morphological changes associated with apoptosis [27]. Recent findings have shed light into detailed interaction models between these proteins that help provide new insights for targeting this pathway (Fig. 1) [5].

The mechanism by which BH3-only protein promote apoptosis is still a matter of intense debate. There is strong evidence for a “direct” model, which suggests that some BH3-only proteins can directly interact with BAX/BAK to induce conformational changes that leads to their activation [28–30]. These so called “activators” include BIM and BID and more recently PUMA and NOXA have shown to act as activators, although with lesser efficacy [31–34]. The other BH3 proteins such as BAD function as “sensitizers” in this model as they mainly interact with anti-apoptotic BCL-2 proteins to release the “activator” BH3-only proteins [31]. The anti-apoptotic BCL-2 proteins can inhibit apoptosis by two modes: either by directly engaging with and inhibiting activation of BAX/BAK or by sequestering activator BH3-only proteins, both of which prevent oligomerization of BAX and BAK (Fig. 1) [35]. Another “indirect” model proposes that the BH3-only proteins do not directly interact with BAX/BAK but instead act by binding to and neutralizing the anti-apoptotic proteins to release BAX/BAK and allow pore formation [36, 37]. A recent study employed genome editing to delete all BH3-only proteins to generate an apoptosis resistant cell line. Interestingly, when MCL-1 and BCL-XL were inhibited in these cells the kinetics of apoptosis was similar to the cells that did express all BH3-only proteins. The authors therefore suggest that BH3-only proteins are not required for the direct activation of BAX and BAK and that they mainly function to repress BCL-XL and MCL-1 [37]. Even if they are not essential for BAX/BAK activation, this does not exclude that BH3 activators can indeed facilitate pore formation through such direct interactions. Regardless of the prevailing model, when the balance is tipped in favor of pro-apoptotic molecules, BAX and BAK will multimerize to form pores in the outer mitochondrial membrane. Although less well studied, BOK was initially identified by its interactions with MCL-1 and is also suggested to have pore forming abilities [38, 39]. However, studies now propose its activation to be regulated by the endoplasmic reticulum-associated degradation pathway, independent of other BCL-2 protein interactions [21, 39, 40].

**Fig. 2:**
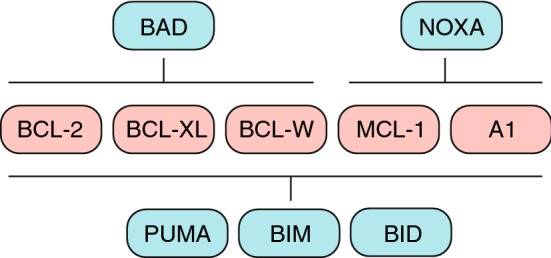
BH3-only proteins have specific affinities for anti-apoptotic proteins

Additional layers of complexity are added to these interaction models as the different members of the BCL-2 family show preferential binding patterns among each other. For instance, BH3-only proteins bind to pro-survival members with different affinities (Fig. 2). BH3-only proteins such as BIM, PUMA and tBID (“activators”) can engage with all anti-apoptotic proteins whereas others are more limited in their interactions [41, 42]. BAD interacts with BCL-2, BCL-XL and BCL-W, while HRK binds specifically to BCL-XL and NOXA shows preferential binding to MCL-1 and A1. Studies also show preferential interactions between activator BH3-only proteins and BAX and BAK. So while BID prefers to interact with BAK, BIM shows either no preference or preference for activation of BAX [30, 43]. An additional layer of complexity is introduced by specific interactions between anti-apoptotic proteins and effector proteins. For example, BAK is not inhibited by BCL-2 while MCL-1 and BCL-XL do restrain its activation [44]. Retrotranslocation of BAX and BAK away from the mitochondria into the cytosol is also regulated by the different pro-survival proteins. While BAX is retrotranslocated by BCL-2, BCL-XL and MCL-1, BAK retrotranslocation is only regulated by BCL-XL and MCL-1 [26]. This retrotranslocation process prevents the activation of BAX/BAK at the mitochondrial outer membrane and thereby also prevents pore formation and mitochondrial outer membrane permeabilization (MOMP).

#### Outcomes of MOMP

MOMP is often considered to be the point of no return for apoptosis to occur [45]. Once the outer membrane is permeabilized by BAX/BAK pore formation, soluble proteins such as cytochrome c are released from the mitochondrial intermembrane space into the cytosol [46–48]. Here cytochrome c binds to APAF-1 to form the apoptosome, which activates caspase-9 and ultimately leads to cleavage and activation of effector caspases such as caspase-3 or 7. MOMP also leads to the release of other proteins such as second mitochondria derived activator of caspase (SMAC) which releases caspase-3 from inhibitory proteins such as X-linked inhibitor of apoptosis (XIAP) [49]. The effector caspases are responsible for the cleavage of various proteins and eventual dismantling of the cell into apoptotic bodies. Despite being the executors of apoptosis, prevention of caspase activation does not always circumvent apoptosis. Permeabilized mitochondria can still lead to a cell’s death in the absence of caspases due to mitochondrial distress, a form of death termed caspase-independent cell death [50–52]. Interestingly, several studies report cell survival despite caspase activation [53–55]. When only a minority of mitochondria commit to MOMP, cells may prevent cell death and maintain their clonogenic potential [56]. In fact, the limited caspase activation resulting from this so called “minority MOMP” can induce caspase associated DNAse activity and thereby DNA damage. Cells that survive despite activation of the apoptotic pathway by such mechanisms may thus become genetically unstable and potentially tumorigenic [56–58].

### Mechanisms of apoptotic de-regulation

De-regulated apoptosis is observed in most tumor types and is considered one of the hallmarks of cancer. Evasion of apoptosis allows tumor cells to bypass oncogene-induced cell death and can also promote sustained tumor growth, survival during metastatic spread and therapy resistance. De-regulation of the BCL-2 family not only occurs during tumorigenesis and outgrowth but is also observed as part of the tumor evolution that takes place in response to therapy [59–61]. The intrinsic pathway of apoptosis is often modified to tip the balance towards reduced apoptosis by altering one or both of the two main components of the pathway.

Not surprisingly, an increased expression of pro-survival BCL-2 proteins is found in several cancer types. This increase can be achieved by several mechanisms including chromosomal translocations [8], gene amplifications [62], increased transcription [63–65] and post-transcriptional and post-translational modifications (PTM) [66, 67]. Chromosomal gain mediated amplification of *Mcl-1* and *Bcl-xL* were found to be the most frequent alterations across 26 tumor types, particularly in solid tumors [62]. Anti-apoptotic adaptation can also occur through PTMs that enhance the activity of pro-survival proteins [68, 69]. Most tumors generally rely on the up-regulation of one or two anti-apoptotic proteins for resistance, which varies from tumor-to-tumor and even within the same tumor type [70–73]. Thus, most cancers present heterogeneous expression and dependence on anti-apoptotic proteins.

Another mechanism of altering the apoptotic threshold is to decrease the expression or modulate the activity of pro-apoptotic BH3-only proteins. Loss of BH3-only proteins is only mildly oncogenic on its own but can be tumorigenic in certain contexts such as co-occurrence with MYC activation [74]. Loss of P53 occurs in many cancers and leads to the downregulation of its transcriptional targets PUMA and NOXA [75, 76]. Several studies document other tumor-associated changes in BH3-only proteins by various mechanisms including mutation, loss of heterozygosity or epigenetic silencing [77]. Reduced expression or activity of the BCL-2 family effector proteins is also a potent mechanism for apoptosis evasion in tumor cells. BAX somatic frameshift mutations are selected for in microsatellite instable gastric, colon and endometrial tumors [78]. Localization changes can also affect their apoptotic activity as observed in AML [79]. Studies have noted changes in their activity induced by phosphorylation and anti-cancer therapy that affect their pore forming abilities [59, 80]. BOK deletions are quite frequently detected in a range of tumors [62]. However, BOK deficient mice show no overt phenotypic changes and cells derived from these mice are not hampered in apoptosis [81]. Several studies suggest that BOK exerts its anti-tumorigenic effects through non-apoptotic functions [82, 83]. On the other hand, a pro-tumorigenic role for BOK is reported in hepatocellular carcinoma where deletion of BOK is infrequent [84].

### De-regulation of the BCL-2 family in CRC

Anti-apoptotic adaptation is a crucial step in CRC initiation and advancement. An accumulation of alterations that enable apoptosis evasion is observed as CRC progresses from adenoma-to-carcinoma stages. The increased apoptotic threshold hampers the efficacy of various chemotherapeutics and thus presents itself as a valuable target for CRC therapy. Several studies highlight modifications in the intrinsic apoptosis pathway at various stages of the disease. Here we review the role of the BCL-2 family in transformation of a healthy colon into adenomas and examine the pathway’s de-regulation as the disease progresses towards the carcinoma stage.

#### Apoptosis in the normal colon

Homeostasis in the colon is maintained by a balance between proliferation and apoptosis. The colonic epithelium consists of a single layer of epithelial cells that is organized to form invaginations called crypts [85]. Lgr5+ stem cells reside at the bottom of these crypts, which proliferate to give rise to intermediate transit amplifying cells [86]. Transit amplifying cells differentiate into various lineages as they move along the crypt towards the luminal face of the colon where they eventually get shed and die by apoptosis [87]. The entire colon gets renewed within 4–5 days thus making apoptosis an active pathway in intestinal regeneration and maintenance. Two forms of apoptosis occur in the intestine—spontaneous apoptosis and damage-induced apoptosis. Spontaneous apoptosis occurs in general intestinal turnover whereas damage-induced apoptosis is elicited in response to stresses such as irradiation, chemotherapy and pathogens.

Several anti-apoptotic proteins and pro-apoptotic effectors are expressed in the colon and have important functions in both forms of apoptosis (Table 1). Studies on BCL-2 expression in the colon all conclude that it is mainly localized in the bottom of the crypt, where the stem cells reside [88]. On the other hand, BCL-XL and MCL-1 show more general expression in the normal intestine, not localized to a particular crypt compartment but specifically showing an apical staining pattern in cells [89, 90]. BCL-W was hardly detectable in human normal tissue however a study in mice did show expression in the intestine [91, 92]. BAX and BAK expression in the crypts is more pronounced in the upper 2/3rd part than the bottom, in contrast to pro-survival BCL-2 [90, 93]. These expression studies support the observed pattern of proliferation in the bottom of the crypt and apoptosis at the top.

**Table 1:** Expression alterations of BCL-2 family members in CRC progression from adenoma-to-carcinoma compared to healthy epithelium

BCL-2 family protein	Normal	Adenoma	Adenocarcinoma	References
BCL-2	Expressed	Increase	Decrease	[94–99]
			Increase	[100, 101]
		Decrease	Decrease	[102]
		No change	Increase	[103, 104]
BCL-XL	Expressed	Increase	Increase	[90, 102, 104, 105]
BCL-W	Not expressed	Not expressed	Increase	[92]
MCL-1	Expressed	Increase	Decrease	[102]
		Decrease		[104]
BAX	Expressed	No change	No change	[90, 102]
BAK	Expressed	Decrease	Decrease	[90, 102]
PUMA	Expressed	-	Increase	[106]
NOXA	Expressed	-	No change	[107]
BID	Expressed	-	Increase	[108, 109]

Knockout (KO) mouse models of pro- and anti-apoptotic proteins provide insight into their role in spontaneous and damage-induced apoptosis in the colon. Homozygous BCL-2 null mice show increased spontaneous apoptosis compared to wild-type mice in the crypt bottom of the colon [88]. This however was not observed in the small intestine of BCL-2 KO mice, which show no overt phenotypic changes [88, 110]. An increase in damage-induced apoptosis upon irradiation and 5-FU treatment is also observed in the colonic stem cells of BCL-2 KO mice [111]. Intestinal specific BCL-XL KO mice do not present with any changes to spontaneous apoptosis, thus maintaining colon integrity [104]. Similarly, knocking out BCL-W does not affect spontaneous intestinal apoptosis. However, these KO mice do display an important role for BCL-W in damage-induced apoptosis, particularly in the small intestine [91]. Both spontaneous and damage-induced apoptosis are unaffected by BAX KO, while the colon in BAK-null mice is significantly affected with crypt hyperplasia and reduced damage-induced apoptosis [111, 112]. The colon of BAK KO mice present with an increase in goblet cell numbers and a decrease in endocrine populations resulting from reduced basal apoptosis levels [112]. This suggests a unique role for BAK in regulating intestinal homeostasis that is not interchangeable with BAX.

#### The BCL-2 family in intestinal transformation

A progressive inhibition of apoptosis occurs during CRC progression [103]. This is particularly crucial during initiation where the tumor cell needs to overcome the apoptotic check in response to oncogenic signaling. Immunohistochemical (IHC) analysis of BCL-2 family protein expression in normal and adenoma tissues suggest that various members are de-regulated upon transformation (Table 1). IHC data indicate that colon adenomas have increased levels of anti-apoptotic protein BCL-XL while BCL-W is hardly detectable [92, 102, 104]. BCL-2 and MCL-1 stainings show inconsistent results in different studies. While several studies find an increase in BCL-2 protein levels in adenomas [94–101], there are also reports of either a decrease or no change in expression compared to normal tissue [102–104] (Table 1). However, the expression of BCL-2 specifically in the stem cells of the healthy colon has an important role in tumorigenesis [110]. Deletion of APC leads to adenoma formation in the mouse small intestine due to hyperactivation of the Wnt pathway. In stem cell-specific BCL-2 null mice, loss of APC gives rise to significantly fewer adenomas, thus indicating that BCL-2 is crucial for tumor initiation and survival [110]. Another study suggests an important role for BCL-XL in CRC development where intestinal epithelial cell-specific BCL-XL KO mice develop fewer tumors in an inflammation-driven tumor model [104]. Similarly, PUMA loss facilitates CRC progression as PUMA KO mice develop more adenomas in both the APC Min/+ and inflammation-driven tumor models [113]. As PUMA is driven largely by a p53 response, this suggests that oncogenic stress elicited by APC deletion drives a p53-dependent apoptotic response. IHC expression data of the effector proteins suggest that levels of pro-apoptotic protein BAX remain unchanged, while BAK is clearly decreased in adenomas (Table 1) [90, 102]. The observed downregulation of BAK could in part be due to oncogenic KRAS signaling. Ectopic re-expression of BAK in KRAS-transformed intestinal cells reduces their tumorigenicity [114]. More recently, a lesser known member of the BCL-2 family, namely BCL-G, was found to play a crucial role in colon tumorigenesis [115]. BCL-Gs (splice variant) only has the BH3 domain and has pro-apoptotic activity as it can bind to and inhibit BCL-XL [116]. Loss of BCL-G resulted in accelerated tumor formation in an inflammation-driven tumor model but not in the APC Min/+ mouse model [115]. Importantly, it is unclear whether this relates to its apoptotic functions as deletion affects the Mucin structure of the mucosal layer, indicating a non-apoptotic role for BCL-G in this context.

#### The BCL-2 family in CRC carcinogenesis

CRC develops in a step-wise manner with sequential accumulation of specific genetic mutations that dictate the progression from adenoma-to-carcinoma stages [117]. This progression is accompanied by several changes in the apoptotic threshold of the cancer cells with an overall inhibition of apoptosis [103]. Most members of the BCL-2 family show altered expression patterns in CRC tumors, which plays a role in cancer progression and therapy resistance.

Of the anti-apoptotic proteins, BCL-2 and MCL-1 expression is found to be decreased in CRC while BCL-XL and BCL-W show increased expression (Table 1). While BCL-2 plays a key role in adenoma formation, a progressive decrease in its expression during tumor progression indicates less of a role in CRC survival and resistance [90, 94–99, 110, 118]. The mechanism behind this decrease in expression has not been explored but studies suggest reciprocal regulation of BCL-2 and BCL-XL due to their inverse expression patterns [102]. Of all cancer types, *Bcl-xL* is amplified most often in cancers of the colon and a vast majority of CRCs present with BCL-XL overexpression [90, 102, 104, 105, 119]. This increased expression has been shown to be crucial for CRC survival and progression [90, 104, 119]. Colon cancer stem cells (CSCs) in particular are considered to be the chemo-refractory sub-population in primary CRC cultures and this resistance is mainly BCL-XL dependent [120]. Indeed, overexpression of BCL-XL in these cultures renders the differentiated population chemo-resistant [120]. De-regulation of anti-apoptotic MCL-1 also plays a crucial role in CRC chemo-resistance. While MCL-1 expression studies suggest decreased levels in CRC, it is important to note that its protein turnover rates are quite high with a half-life of approximately 30 min [121, 122]. Phosphorylation of MCL-1 by GSK3β facilitates binding of FBW7, an E3 ubiquitin ligase which ubiquitylates and causes proteasomal degradation of MCL-1 [122, 123]. This degradation of MCL-1 is necessary for the efficacy of various targeted therapeutics against CRC cell lines [124]. Alterations in proteins regulating this degradation process often occur in CRC. Inactivating FBW7 mutations are found in CRC cell lines which promote MCL-1 stability and therefore, resistance to chemotherapy [123]. USP9X, a deubiquitinase enzyme that is reported to deubiquitinate and thus stabilizes MCL-1 protein levels, is also higher expressed in CRC cell lines [125].

In addition to the de-regulation of anti-apoptotic BCL-2 family members, several studies indicate altered expression and activity of the apoptosis effector proteins in CRC. BAK expression levels are decreased in colorectal tumors, but in most cases BAX levels appear to be unchanged [90, 102]. However, mutation analyses reveal the incidence of *Bak* mutations to be relatively low in CRC, but on the other hand, *Bax* frameshift mutations are quite frequently selected for in microsatellite instable tumors and this results in reduced apoptosis in BAX negative tumors [126–128]. The pro-apoptotic protein BAX interacting factor-1 (BIF-1) is found to be decreased in CRC tissues which might also result in reduced efficacy of BAX in CRC [129]. A recent study indicates a role for BOK in chemo-resistance in CRC as primary patient-derived organoids that are 5-FU resistant show decreased BOK expression compared to 5-FU sensitive organoids. This role is independent of its pro-apoptotic activity as the study shows BOK regulates uridine metabolism and thereby 5-FU chemo-conversion, thus a decrease in its expression promotes 5-FU resistance [130].

Of the pro-apoptotic BH3-only proteins, occasional mutations have been reported for *Bad*, but no mutations were detected in *Noxa*, *Puma* and *Bik* in CRC tumors [106, 107, 131, 132]. 293T cells transfected with tumor-associated mutant BAD show decreased apoptosis induction and mutant BAD binds inefficiently to BCL-2 and BCL-XL [132]. Although mutations are infrequent, altered expression of BH3-only proteins is frequently observed in CRC. The promoter region of *Hrk* is found to be methylated in some CRC cases and treatment of cell lines with de-methylating agents re-introduces *Hrk* expression and sensitivity to chemotherapy [133]. An increased expression of PUMA and BID is detected in CRC, while NOXA levels remain unchanged (Table 1) [106–109]. This increase in pro-apoptotic proteins might be counter-intuitive for apoptosis evasion but most likely, such mechanisms are employed by a cell as a protective measure against transformation. Therefore, this increased expression could indicate the presence of compensatory mechanisms that enable tumor cells to override such selection pressures. PTMs that de-regulate BH3-only proteins are also reported in CRC such as an increase in BAD phosphorylation, which attenuates BAD pro-apoptotic activity and facilitates tumorigenesis [106, 134, 135].

Considering the frequent alterations in the BCL-2 family that occur in CRC, various studies have assessed the potential of different members of the family as prognostic biomarkers for the disease [136]. However, due to the complex interactions of the various regulators of this pathway, none of the proteins on their own could reliably predict clinical response to therapy [136]. For this reason, a recent study employed a computational model that reflects the dynamic regulation of MOMP by the BCL-2 proteins to successfully identify high-risk CRC patients [137]. This highlights the importance of the various interactions among the BCL-2 family members that play a major role in determining tumor progression and therapy response.

### Targeting the BCL-2 family

**Fig. 3:**
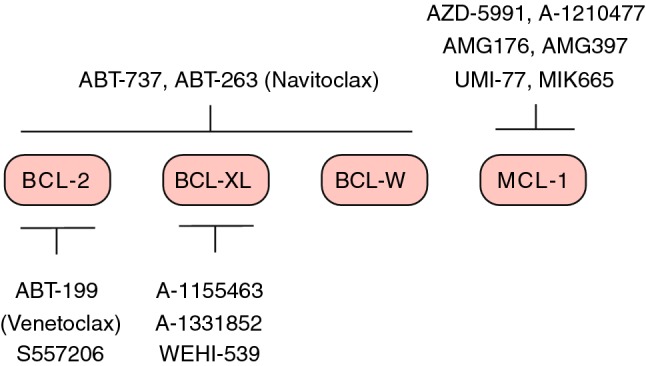
An overview of selective BH3 mimetics designed to inhibit anti-apoptotic proteins

Cancer cells adapt to the various pressures of oncogenic transformation such as checkpoint evasion, metabolic stress and replicative damage by de-regulating the BCL-2 family and becoming more refractory to apoptosis. In order to handle these stress signals, the cancer cell is in a precarious position where it requires this apoptotic block to hold. This dependence on the BCL-2 family presents a therapeutic window of opportunity where pushing this block can specifically target cancer cells for apoptosis. Highly specific small molecule inhibitors called BH3 mimetics have therefore been developed to target anti-apoptotic BCL-2 proteins by mimicking the action of BH3-only proteins (Fig. 3). Here we review the reported efficacy of these inhibitors and examine their potential for CRC therapy.

#### BH3 mimetics: pushing the apoptotic block

Early efforts to overcome the anti-apoptotic defense mechanism in cancer were mainly driven by screening libraries of natural products, which yielded several classes of compounds that can inhibit anti-apoptotic BCL-2 proteins [138]. Two of these, AT-101 and Obatoclax (GX15-070), have been tested in clinical trials for solid and hematological malignancies (see clinicaltrials.gov). Although these compounds can inhibit multiple anti-apoptotic BCL-2 members, they do not show sufficient clinical efficacy to warrant their approval [138, 139]. Using structure-based design to selectively target anti-apoptotic proteins proved successful with the generation of ABT-737, the first BH3-mimetic to be developed [140]. Designed using NMR-based screening, this compound mimics the binding pattern of BAD to inhibit the hydrophobic groove of BCL-2, BCL-XL and BCL-W. For clinical use, an orally bioavailable analog of ABT-737 was developed named ABT-263 or Navitoclax [141]. Notwithstanding the remarkable efficacy shown by this drug in pre-clinical studies [141–144], its use in the clinic is hampered by severe platelet toxicity [145]. Inhibition of BCL-XL in particular is found to be the cause of the observed thrombocytopenia, revealing its role in regulating platelet lifespan [146]. However, appropriate dosing strategies can help control the thrombocytopenia and indeed, several clinical trials testing the safety and efficacy of ABT-263 in a range of tumor types are ongoing (see clinicaltrials.gov) [139]. Another dual BCL-2 and BCL-XL inhibitor, AZD4320 shows potent tumor regression in vivo in hematological and solid malignancies [139]. This drug, re-formulated as a novel nanomedicine (AZD0466), is currently in a phase I clinical trial for both blood and solid tumors (NCT04214093). APG-1252 is another newly developed inhibitor of BCL-2 and BCL-XL that shows potent activity in vivo in small cell lung cancer models and triggers less platelet toxicity compared to Navitoclax [147]. The authors claim that this is because the drug on its own is poorly permeable but is converted to an active metabolite particularly in the tumor cells. This metabolite effectively reduces tumor growth while hardly being detected in the plasma, thereby avoiding toxicity [147]. Further study is needed and will come from phase I clinical trials, which are currently ongoing for lung cancer and other solid tumors in several countries (NCT03387332, NCT03080311, NCT04001777) [148].

To circumvent the observed platelet toxicity of inhibiting BCL-XL, it was hypothesized that specifically targeting BCL-2 could provide a wider therapeutic window with fewer toxicities. This led to the development of ABT-199 or Venetoclax, the first BH3 mimetic to receive FDA approval for relapsed/refractory chronic lymphocytic leukemia (R/R CLL) [149]. This landmark drug registration was followed by two more FDA approvals for Venetoclax in combination therapy for patients with R/R CLL and treatment naïve acute myeloid leukemia (AML) [139]. More than 200 clinical trials, particularly for hematological malignancies, have since been conducted or are ongoing (see clinicaltrials.gov) [150]. S55746 is another BCL-2 specific inhibitor which demonstrates significant efficacy against BCL-2 dependent tumors in vitro and in vivo and has been tested in two clinical trials (NCT02920697, NCT02603445) [139]. The results of the dose-escalation study have been recently released which show that the trial had to be terminated as the target active exposure of the drug in patients was not reached.

Studies of Venetoclax in solid tumors are limited with so far a trial in combination with tamoxifen for estrogen receptor positive breast cancers (ISRCTN98335443) and three others in a range of solid tumors (NCT03000257, NCT04029688, NCT03082209). These trials will test Venetoclax efficacy in combination with diverse compounds, which include the trail receptor agonist ABBV-621, MDM2 inhibitor Idasanutlin and anti-PD1 monoclonal antibody ABBV-181. Although there are promising indications for BCL-2 inhibition in solid tumors, anti-apoptotic adaptation is more closely associated with the overexpression of BCL-XL [151]. Several BCL-XL inhibitors have been designed and the first specific inhibitor, WEHI-539, binds to BCL-XL with high selectivity and affinity [152]. Since then, further efforts led to the design of A-1155463 and A-1331852, two far more potent inhibitors of which the latter is also orally bio-available [153, 154]. Studies show promise for these inhibitors alone and in combination for treatment of solid tumors [154, 155]. A-1331852 enhances the efficacy of docetaxel in a range of solid tumors including breast cancer, NSCLC, and ovarian cancer both in vitro and in vivo [155]. In addition to this efficacy, BCL-XL inhibition does not result in neutropenia, which is a common toxicity of Venetoclax, shown to result from BCL-2 inhibition specifically [155]. While both these inhibitors induce platelet toxicity in vivo, this toxicity is reversible and therefore could be overcome by proper dosing strategies [154, 155]. These promising inhibitors are yet to enter clinical trials.

In the wake of inhibitors targeting BCL-2 and BCL-XL, MCL-1 is emerging as an increasingly promising target given its role in malignant cell survival and resistance to various anti-cancer therapies. Several tumor types show heterogeneous dependence on MCL-1 including breast, lung, multiple myeloma (MM) and MYC-driven lymphomas [139]. Interestingly, there is evidence of MCL-1 expression levels increasing upon treatment with ABT-737 and this is implicated in resistance to other BH3 mimetics such as Navitoclax and Venetoclax, which do not inhibit MCL-1 [156, 157]. MCL-1 is also involved in resistance to various chemotherapeutics, making it a promising target for combination and second-line treatment in resistant tumors [158, 159]. MCL-1 has proven far more difficult to target and only recently, successful specific inhibition has been achieved by BH3 mimetics such as A-1210477, UMI-77, S63845, AZD-5991 and AMG176 [160]. A-1210477 shows in vitro efficacy against a range of cancer cell lines while UMI-77 is effective in vitro and in vivo against pancreatic and breast cancers [161, 162]. AMG176 is the first MCL-1 inhibitor to enter a clinical trial for MM and AML (NCT02675452) [163]. It is also being tested in combination with Venetoclax for AML and non-Hodgkin’s lymphoma (NHL) (NCT03797261). A similar Amgen MCL-1 inhibitor, AMG-397, is also being tested in the clinic for MM, AML and NHL (NCT03465540) [160]. However, trials for both these inhibitors have been recently suspended due to potential cardiac toxicity. Another MCL-1 specific inhibitor AZD-5991 shows significant anti-tumor activity in vivo with complete regression in mouse models of MM and AML, based on which a phase I clinical trial has been set up for patients with hematological malignancies (NCT03218683) [164]. A highly potent derivative of S63845 called S64315/MIK655 shows substantial in vivo activity against MM, AML and MYC-driven lymphomas and is also being tested in clinical trials for these malignancies as a monotherapy (NCT02992483, NCT02979366) and in combination with Venetoclax (NCT03672695) [165].

The approval of Venetoclax has heralded a new era of significant progress in therapeutic targeting of anti-apoptotic BCL-2 proteins (Fig. 3). A major challenge in the clinical application of these inhibitors is to predict the appropriate anti-apoptotic molecules to inhibit in a context-dependent and perhaps even a personalized manner. Dynamic BH3 profiling is one such technique that not only predicts the anti-apoptotic dependencies of a tumor, but also predicts response to various chemotherapeutics [18]. In this technique, control vs. drug-treated tumor cells are incubated with BH3 peptides that have specific anti-apoptotic binding partners (Fig. 2). Subsequent retention of cytochrome c is measured to assess the overall efficacy of the compound and the possible anti-apoptotic proteins that the tumor depends on. Such insight can not only predict treatment response in patients, but also greatly benefit the rationalized use of BH3-mimetics for treatment. Overall, these inhibitors not only show great promise for cancer therapy but also serve as invaluable tools for understanding the ever-changing anti-apoptotic landscape of this disease.

#### BH3 mimetics for CRC therapy

While there are no CRC specific clinical trials testing the efficacy of BH3 mimetics, there is compelling evidence for the use of these inhibitors in CRC treatment. De-regulated expression of anti-apoptotic proteins is observed in all stages of CRC development and these changes can guide the use of BH3 mimetics for treatment. A recent study conducted in a panel of diverse cell lines finds that BCL-2 dependence directly correlates with BCL-2 expression levels, such that high expressers are more sensitive to ABT-199 treatment [166]. BCL-2 is expressed in crypt stem cells and plays a critical role in facilitating intestinal stem cell transformation and adenoma survival [110]. Treatment with ABT-199 while intestinal stem cells are undergoing APC deletion and hence transformation strongly impairs adenoma outgrowth, which suggests that BCL-2 is a potential target for CRC chemoprevention in patients with high risk conditions such as familial adenomatous polyposis [110]. However, BCL-2 expression is gradually lost as the disease progresses and most CRC cell lines are insensitive to ABT-199 [166]. Colon CSCs, which are particularly chemo-resistant, no longer express any BCL-2 and do not respond to ABT-199 treatment [120].

Considering the high levels of BCL-XL found in most CRC tissues, it has been a target of prime interest for CRC treatment [90, 104, 119]. Indeed the same colon CSCs that do not respond to BCL-2 inhibition are very sensitive to BCL-XL specific inhibitor WEHI-539, which impairs CSC clonogenicity and enhances the efficacy of chemotherapy [120]. The dual inhibitor ABT-737 also potently induces cell death in these CSCs while also showing efficacy on CRC ex vivo tissue and cell lines in combination with diverse chemotherapies [104, 120, 167, 168]. Several studies indicate that the efficacy of ABT-737, or its orally bioavailable counterpart ABT-263, in solid tumors is primarily due to the inhibition of BCL-XL [104, 120, 155]. The efficacy of BCL-XL inhibition is closely associated with MCL-1 activity, in particular predicted by NOXA expression which specifically inhibits MCL-1 [166]. Cells that express high level of NOXA either basally or induced by other therapies show increased sensitivity to BCL-XL inhibition by A-1155463 and ABT-737 [119, 169]. This suggests that concurrent treatment with MCL-1 inhibitors might potentiate the effect of BCL-XL inhibition in tumors that either present with high MCL-1 or low NOXA levels [170, 171]. In a CRC cell line HCT116, treatment with A-1155463 alone was sufficient to induce apoptosis, while MCL-1 inhibitor S63845 alone did not induce any apoptosis. However, combining the two compounds resulted in more pronounced apoptosis even in the absence of all BH3-only proteins, in a BAX dependent manner [171].

Taken together, these pre-clinical data suggest that BH3 mimetics hold great promise for CRC treatment. Early stage high risk adenomas that express BCL-2 might benefit from BCL-2 inhibition with ABT-199, thus preventing tumor progression. On the other hand treatment of more advanced carcinomas with BCL-XL specific inhibitors, perhaps in combination with MCL-1 inhibition might prove more effective. The main hindrance for the use BCL-XL inhibitors in clinical practice is the observed thrombocytopenia, as BCL-XL regulates platelet lifespan [146]. This can be managed by appropriate dosing strategies, particularly by using lower doses of the inhibitors in synergistic combinations with other chemotherapeutics [139]. Pre-clinical studies that test such possibilities would greatly aid the advancement of the use of BH3 mimetics in CRC therapy.

## Conclusions and future perspectives

Recent findings have provided novel insights into the interactions of the BCL-2 family members that regulate the intrinsic pathway of apoptosis. Although the intricacies of this regulation are complex and still a matter of intense debate, it is clear that this family of proteins has an important role to play in homeostasis and tumorigenesis in the colon. De-regulation of the BCL-2 family occurs through various mechanisms and many CRC tumors show dependence on different anti-apoptotic proteins through different stages of the disease, making them a promising target for therapy. BH3 mimetics show potent induction of apoptosis in vitro and in vivo in various models. While BCL-2 inhibition can inhibit early adenoma outgrowth, CRC tumors respond well to BCL-XL specific inhibition, more so in combination with Mcl-1 inhibition. Implementing strategies to reduce the toxicities associated with these inhibitors and determining the appropriate BH3 mimetic to administer in a context-dependent and personalized manner (for example with dynamic BH3 profiling) could help untap the full potential of these novel inhibitors in CRC treatment.
